# A variational principle for fluid sloshing with vorticity, dynamically coupled to vessel motion

**DOI:** 10.1098/rspa.2018.0642

**Published:** 2019-04-17

**Authors:** H. Alemi Ardakani, T. J. Bridges, F. Gay-Balmaz, Y. H. Huang, C. Tronci

**Affiliations:** 1 Department of Mathematics, University of Exeter, Penryn Campus, Cornwall TR10 9FE, UK; 2 Department of Mathematics, University of Surrey, Guildford GU2 7XH, UK; 3 Laboratoire de Météorologie Dynamique, École Normale Supérieure and CNRS, Paris 75231, France; 4 Numerical Methods Division, Max Planck Institute for Plasma Physics, Garching 85748, Germany

**Keywords:** nonlinear waves, geometric mechanics, stream functions, rigid-body motion

## Abstract

A variational principle is derived for two-dimensional incompressible rotational fluid flow with a free surface in a moving vessel when both the vessel and fluid motion are to be determined. The fluid is represented by a stream function and the vessel motion is represented by a path in the planar Euclidean group. Novelties in the formulation include how the pressure boundary condition is treated, the introduction of a stream function into the Euler–Poincaré variations, the derivation of free surface variations and how the equations for the vessel path in the Euclidean group, coupled to the fluid motion, are generated automatically.

## 1. Introduction

Variational principles for fluid sloshing, that is free surface flow in an enclosed container, abound, having first been derived independently by Lukovsky [1] and Miles [2], based on Luke's variational principle [3]. Variational principles have since been widely used in the analysis and modelling of fluid sloshing (e.g. Faltinsen & Timokha [4], ch. 2, 7 and references therein). However, in all this work the fluid motion is assumed to be irrotational. A variational principle for fluid sloshing with vorticity was first introduced by Timokha [5] using Clebsch variables to represent the velocity field. In two space dimensions, Clebsch variables represent the vorticity field exactly, but they are difficult to work with and have singularities in the potentials even when the velocity field is smooth (e.g. [6,7]).

In this paper, a new variational principle for fluid flow with a free surface and vorticity is obtained in two dimensions by representing the velocity field in terms of a stream function and using constrained variations. Moreover, the variational principle captures dynamic coupling with the vessel motion; that is, the variational principle produces both the exact fluid equations in a moving vessel, as well as coupling with the exact equations of motion for the vessel. The vessel motion is a path in the special Euclidean group, SE(2), consisting of rotations and translation in the plane.

Variational principles for the coupled motion have been given before, the first by Lukovsky (see [8] and references therein), for the fluid–vessel coupling, and more recently by Alemi Ardakani [9,10], which, in the latter case, includes coupling between the vessel and both interior and exterior fluid motion. However, in all these cases, the fluid motion is taken to be irrotational. Here the aim is to dynamically couple vessel motion to interior fluid flow with an exact and complete representation of the vorticity field.

Variational principles for Eulerian fluid motion with vorticity are notoriously difficult. On the other hand, variational principles in the Lagrangian particle path (LPP) formulation are the natural continuum versions of Hamilton's principle in classical mechanics and are therefore relatively straightforward. For example, Hsieh [11] presents a variational principle for the LPP formulation with a free surface, based on the kinetic minus the potential energy, which is used to model bubble dynamics, and no special constraints or constrained variations are required. Yamada [12] develops a numerical method, based on a Lagrangian variational principle, for sloshing in the LPP setting. However, in general the LPP formulation is not as useful in practice as the Eulerian fluid representation.

In the Eulerian representation, the fluid velocity **u**(**x**, *t*) is studied as a function of position and time. It is the observational approach and the most widely used representation in fluid dynamics. When forecasting the wind in a city, for example, one wants to predict the velocity at a fixed spatial position as a function of time. One is not normally interested in where the parcel of fluid was the day before or the week before. This latter view, where individual fluid parcels are followed for all time, is the LPP description. The position of a parcel of fluid is given by

1.1 x=φ(a,τ),a∈B,

where *τ* = *t* is the representation of time in the Lagrangian setting. Here $B\subset R^{n}$ (spatial dimension *n*) is a fixed reference space that may be the initial configuration of the fluid, *φ*(**a**, 0) = **a**∈*B*. The Lagrangian velocity field follows a fluid particle:

1.2 $$u^{Lag}(a,\tau )=\dot{\varphi }(a,\tau ),$$

where the dot denotes differentiation with respect to *τ* (time derivative with **a** fixed). The two fields are related by

1.3 $$u^{Lag}(a,\tau )=u(\varphi (a,\tau ),t)\;(\text{with}\;t=\tau ).$$

In fact, this identity will be important in transforming natural variational principles in the LPP setting to a variational principle in the Eulerian setting.

Natural variational principles, starting with the kinetic minus the potential energy, fail in the Eulerian setting. For example, take the two-dimensional fluid domain to be in the region 0 < *y* < *h*(*x*, *t*), where *y* = *h*(*x*, *t*) represents the free surface for 0 ≤ *x* ≤ *L*. Then the natural Lagrangian, based on the kinetic minus potential energy, when the velocity is represented by a stream function, is

1.4 $$\delta \int_{t_{1}}^{t_{2}}L(\psi ,h)\;dt=0\;\text{with}\;L(\psi ,h)=\int_{0}^{L}\int_{0}^{h}\left[\frac{1}{2}\rho (\psi_{x}^{2}+\psi_{y}^{2})-\rho gy\right]dx\;dy,$$

where *ρ* is the constant fluid density and *g* is the gravitational constant. Taking free variations with respect to *h* and *ψ* and setting δL/δψ and δL/δh to zero does not lead to the correct governing equations or boundary conditions. The reason being that *ψ* and *h* are not the Lagrangian variables of the problem, and hence the classical Hamilton principle with free variations does not apply when such variables are used.

One thus needs to consider the *constrained variations* induced by the free variations of the Lagrangian variables. For fluids with a fixed boundary, this is known as the *Euler–Poincaré framework* (e.g. Holm *et al.* [13, ch. 11]). In the Euler–Poincaré framework, the constrained variation of the velocity field is given by

1.5 $$\delta u=z_{t}+[u,z],$$

where **z** is a vector-valued free variation, and [ · , · ] is the Lie bracket of vector fields [13,14] (that is, [**u**, **z**] = **u** · ∇**z** − **z** · ∇**u**). However, we will need to introduce two extensions of this theory: firstly, inclusion of a free boundary *h*(*x*, *t*) and an appropriate variation *δh*, and, secondly, how to induce a constrained variation, *δψ*, for the stream function.

The Euler–Poincaré framework was first extended to free boundary flows in Gay-Balmaz *et al.* [15] with a compressible fluid in the interior. The incompressible case can be obtained *a posteriori* by setting density to be constant. Here, the new strategy is to address the incompressible case directly by working with divergence-free vector fields from the start, parametrized with a stream function, then use (1.5) and a Lie algebra homomorphism to obtain *δψ* directly,

1.6 $$\delta \psi =w_{t}+\{w,\psi \},$$

where *w*(*x*, *y*, *t*) is scalar valued and a free variation, and { · , · } is the standard (*x*, *y*)-Poisson bracket for scalar valued functions. This variation, and a reduction from the LPP setting to the Eulerian setting, induces a variation at the free surface

$$\delta h=-W_{x}\;\mathrm{and}\;W(x,t):=w(x,h(x,t),t).$$

The expressions for *δψ* and *δh* are proved in §6. They are derived from first principles using reduction from the LPP to Eulerian formulation and the formula (1.3).

The difficulties with the free boundary are compounded by the use of a stream function formulation: the pressure no longer appears explicitly rendering the dynamic boundary condition at the free surface problematic. This problem is resolved in a novel way by showing that the pressure boundary condition is equivalent to the kinematic conservation law of Gavrilyuk *et al.* [16] (hereafter GKK conservation law), extended to the case of free surface flow relative to a moving frame. Then a key result of the variational construction is how the GKK conservation law emerges from the variational principle, justifying this new form for the pressure boundary condition.

The variational principle is useful for establishing structure, identifying conservation laws, constructing numerical schemes and developing approximate methods such as the multimodal expansion of solutions. Some implications and applications are discussed in §7.

An outline of the paper is as follows. Firstly, the governing equations for the coupled problem are written down in §2. Then a Lagrangian density is formulated based on the kinetic minus potential energy of the fluid and vessel motion, relative to a moving frame in §3. Variations are then taken in the directions *δh*, *δψ*, *δ***q** and δR, where **q** is the body translation vector in two dimensions and R is a rotation matrix in the plane representing the body orientation. Special cases of the resulting equations are given in §5. The justification of the expressions for the constrained variations is given in §6 based on reduction from the LPP setting to the Eulerian setting. In §7 and the concluding remarks section §8 some implications and potential extensions of the new variational formulation are discussed.

## 2. Governing equations

The fluid is incompressible and of constant density *ρ*. The fluid occupies the two-dimensional region

2.1 $$D:=\{(x,y)\in R^{2}:0<y<h(x,t)\;\text{and}\;0<x<L\},$$

for some *L* > 0 and *y* = *h*(*x*, *t*) is a graph representing the free surface, and it is to be determined. The fluid equations are relative to the body-fixed frame with coordinates **x** = (*x*, *y*) and its relation to the spatial frame is given below in (2.6).

Upon denoting the velocity field by **u** = (*u*, *v*), the governing equations for the velocity and pressure, relative to the body frame, are

2.2 $$\begin{array}{rl} \frac{Du}{Dt}+\frac{1}{\rho }\frac{\partial p}{\partial x} & =-g\sin\theta +2\dot{\theta }v+\ddot{\theta }y+{\dot{\theta }}^{2}x-{\ddot{q}}_{1}\cos\theta -{\ddot{q}}_{2}\sin\theta \\ \;\mathrm{and}\;\frac{Dv}{Dt}+\frac{1}{\rho }\frac{\partial p}{\partial y} & =-g\cos\theta -2\dot{\theta }u-\ddot{\theta }x+{\dot{\theta }}^{2}y+{\ddot{q}}_{1}\sin\theta -{\ddot{q}}_{2}\cos\theta , \end{array}\}$$

where *g* > 0 is the gravitational constant and (D*f*/D*t*): = (∂*f*/∂*t*) + *u*(∂*f*/∂*x*) + *v*(∂*f*/∂*y*). The functions (*θ*, *q*_1_, *q*_2_) represent the orientation and translation of the body. These equations are derived in [9] and in §2 of [17]. Conservation of mass relative to the body frame is

2.3 $$u_{x}+v_{y}=0,$$

which also acts as an equation for the pressure. The boundary conditions on the vessel walls are

2.4 u=0at x=0,Landv=0at y=0,

and at the free surface the boundary conditions are

2.5 $$p=p_{a}\;\text{and}\;h_{t}+uh_{x}=v\;\text{at}\;y=h(x,t),$$

where *p*_*a*_(*x*, *y*, *t*) is a given external pressure field. The free-surface boundary condition on the pressure is obtained by assuming that surface tension effects are neglected and by requiring the pressure to be equal to external pressure field at the surface. The free surface equation for the height is the usual kinematic free surface boundary condition in the Eulerian setting.

The body-fixed frame with coordinates **x** = (*x*, *y*) is related to the space-fixed frame with coordinates **X** = (*X*, *Y* ) by

2.6 X=R(t)x+q(t),

where **q** = (*q*_1_, *q*_2_) represents uniform translation of the frame, and

2.7 $$R(t)=\left[\begin{array}{cc} \cos\theta (t) & -\sin\theta (t)\\ \sin\theta (t) & \cos\theta (t) \end{array}\right].$$

**Figure 1.**
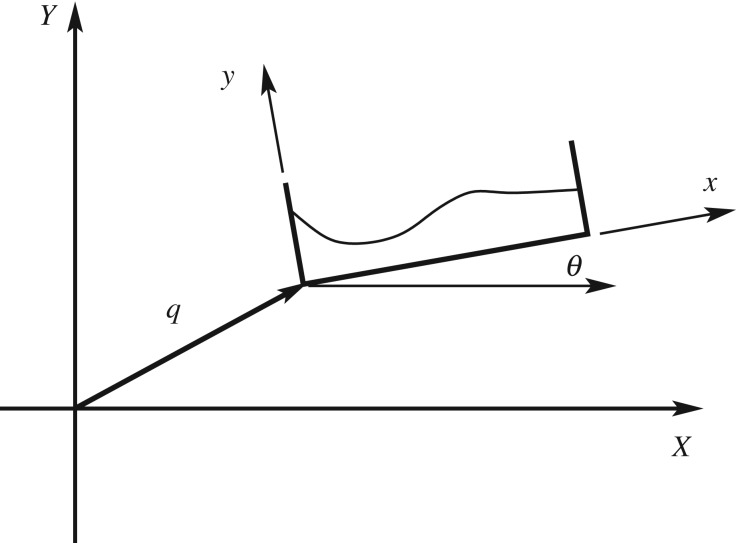
Schematic of the fluid domain relative to the body frame.

A schematic is shown in figure 1. When the axis of rotation is at another point other than the origin of the body axis then (2.6) is replaced by X=R(t)(x+d)+q(t) where **d** is a constant vector. The theory will be developed for **d** = 0, to simplify notation, as the shift in axis of rotation can be added in *a posteriori* as required.

### (a) Stream function and vorticity

In two-dimensional divergence free vector fields in D can be parametrized by a stream function,

2.8 $$u=\psi_{y}\;\mathrm{and}\;v=-\psi_{x}.$$

Conservation of mass (2.3) is satisfied exactly and the vorticity V is defined by

2.9 $$V=v_{x}-u_{y}=-\Delta \psi \;\text{and}\;\frac{DV}{Dt}=-2\ddot{\theta }.$$

The vorticity V is the *relative vorticity*; that is, relative to the body frame. The *absolute vorticity* is $V+2\dot{\theta }$. Substitute the stream function into the free surface boundary condition (2.5),

2.10 $$0=h_{t}+uh_{x}-v=h_{t}+\psi_{y}h_{x}+\psi_{x}=h_{t}+\Psi_{x},$$

where

2.11 Ψ(x,t)=ψ(x,h(x,t),t),

giving the following form for the kinematic free surface boundary condition:

2.12 $$h_{t}+\Psi_{x}=0\;\text{at}\;y=h(x,t).$$

At this point, a difficulty emerges with the pressure boundary condition, *p* = *p*_*a*_ at *y* = *h*, since pressure does not appear in the vorticity-stream function formulation. However, the Euler equations have a gauge symmetry in that an arbitrary function of time can be added to the pressure field without changing the dynamics. Hence the more general boundary condition for the pressure is *p* − *p*_*a*_ = 0 modulo an arbitrary function of time, or

2.13 $$\frac{\partial }{\partial x}p_{a}=\frac{\partial }{\partial x}p(x,h(x,t),t)=p_{x}+p_{y}h_{x}\;\text{at}\;y=h(x,t).$$

Substituting for *p*_*x*_ and *p*_*y*_ from (2.2) then gives

2.14 $$\begin{array}{rl} \frac{Du}{Dt}+h_{x}\frac{Dv}{Dt}+\frac{1}{\rho }\frac{\partial p_{a}}{\partial x} & =-g\sin\theta +2\dot{\theta }v+\ddot{\theta }y+{\dot{\theta }}^{2}x-{\ddot{q}}_{1}\cos\theta -{\ddot{q}}_{2}\sin\theta \\ & \;+h_{x}(-g\cos\theta -2\dot{\theta }u-\ddot{\theta }x+{\dot{\theta }}^{2}y+{\ddot{q}}_{1}\sin\theta -{\ddot{q}}_{2}\cos\theta ), \end{array}$$

at *y* = *h*(*x*, *t*). Although this boundary condition looks complicated it has an elegant and simple form

2.15 $$K_{t}+{\left(uK-\Xi \right)}_{x}=0,$$

with

2.16 $$K=\rho [u-\dot{\theta }h+v_{1}+h_{x}(v+\dot{\theta }x+v_{2})],$$

and flux function

2.17 $$\Xi =\frac{1}{2}\rho {\left(u-\dot{\theta }h+v_{1}\right)}^{2}+\frac{1}{2}\rho {\left(v+\dot{\theta }x+v_{2}\right)}^{2}-\rho (\Gamma_{1}x+\Gamma_{2}h)-p_{a},$$

where ***Γ*** = (*Γ*_1_, *Γ*_2_) and **v** = (*v*_1_, *v*_2_) are the gravity vector and vessel acceleration vector *relative to the body frame* and explicit expressions for them are given in §3.

The conservation law (2.15) is a generalization of the GKK conservation law [16] to the case of free boundary flow relative to a moving frame. When $\theta =\dot{\theta }=v_{1}=v_{2}=0$ and *p*_*a*_ = 0 the above conservation law reduces to

2.18 $$K_{t}+{\left(uK+\rho gh-\frac{1}{2}\rho u^{2}-\frac{1}{2}\rho v^{2}\right)}_{x}=0,\;\mathrm{and}\;K=\rho (u+vh_{x}),\;\text{at}\;y=h(x,t),$$

which is the form of the *kinematic conservation law* given in eqn (14) of [16].

### (b) Summary of the fluid equations

In summary, in the vorticity-stream function formulation, the governing equations (2.2) are replaced by

2.19 $$V=-\Delta \psi \;\text{and}\;\frac{DV}{Dt}=-2\ddot{\theta }\;\text{in}\;D.$$

The boundary conditions at the solid walls are

2.20 $$\psi_{x}=0\;\text{at}\;y=0\;\mathrm{and}\;\psi_{y}=0\;\text{at}\;x=0,L.$$

The boundary conditions at the free surface are

2.21 $$h_{t}+\Psi_{x}=0\;\text{and}\;K_{t}+{\left(\psi_{y}K-\Xi \right)}_{x}=0\;\text{at}\;y=h(x,t),$$

with *K* and *Ξ* given in (2.16) and (2.17), respectively, with *u*, *v* replaced by their stream function representation. The aim is the show that these governing equations for the fluid follow from a variational principle.

### (c) Summary of the vessel equations

The fluid equations in §2b are dynamically coupled to the vessel motion. The vessel position and orientation are given in terms of *θ*(*t*) and **q**(*t*) and they are coupled to the fluid motion. Governing equations for (*θ*(*t*), **q**(*t*)) can be deduced from Newton's Law for the total linear and angular momentum.

The translation of the vessel follows from the conservation of total linear momentum

2.22 $$\frac{d}{dt}\int_{0}^{L}\int_{0}^{h}\rho R(u+\Omega \times x+R^{T}\dot{q})\;dy\;dx=-\int_{0}^{L}\int_{0}^{h}\rho gE_{2}\;dy\;dx.$$

The orientation of the vessel follows from the conservation of total angular momentum

2.23 $$\frac{d}{dt}\left[\int_{0}^{L}\int_{0}^{h}\rho (Rx+q)\times R(u+\Omega \times x+R^{T}\dot{q})\;dy\;dx\right]=-\int_{0}^{L}\int_{0}^{h}\rho g(Rx+q)\times E_{2}\;dy\;dx.$$

In these equations,

$$\Omega =\dot{\theta }e_{3}=\dot{\theta }E_{3}=\omega ,$$

that is the body angular velocity ***Ω*** equals the space angular velocity ***ω***. The basis for the spatial frame is denoted by (**E**_1_, **E**_2_, **E**_3_) and the basis for the body frame is denoted by (**e**_1_, **e**_2_, **e**_3_). The fluid velocities **U** and **u** in the spatial and body frame, respectively, are related by

2.24 $$U=\dot{R}x+Ru+\dot{q}=R\left[u+\Omega \times x+R^{T}\dot{q}\right].$$

The governing equations for the vessel, (2.22) and (2.23), can be expanded and simplified, but it is easier to develop these equations in the form that emerges from the variational principle.

## 3. Lagrangian of the coupled system

The proposed variational principle is

3.1 $$\delta \int_{t_{1}}^{t_{2}}L\;dt=0,$$

where the Lagrangian is the kinetic minus potential energy

3.2 $$L={KE}^{\;f}+{KE}^{v}-{PE}^{\;f}-{PE}^{v},$$

where the superscript *f* indicates fluid and *v* the vessel. These energies are formulated as follows.

The energy densities of the fluid are

3.3 $${KE}^{f}=\int_{0}^{L}\int_{0}^{h}\hat{{KE}^{f}}\;dy\;dx\;\text{and}\;{PE}^{f}:=\int_{0}^{L}\int_{0}^{h}\hat{{PE}^{f}}\;dy\;dx-\int_{0}^{L}\int_{h}^{\infty }p_{a}\;dy\;dx,$$

with

3.4 $$\begin{array}{rl} \hat{{KE}^{f}} & =\frac{1}{2}\rho ∥U{∥}^{2}\\ & =\frac{1}{2}\rho ∥u+\Omega \times x+R^{T}\dot{q}{∥}^{2}\;\text{(using (2.24))}\\ & =\frac{1}{2}\rho \left(u+\Omega \times x+v\right)\cdot \left(u+\Omega \times x+v\right)\\ & =\frac{1}{2}\rho ∥u{∥}^{2}+\rho u\cdot \Omega \times x+\frac{1}{2}\rho ∥\Omega \times x{∥}^{2}+\rho v\cdot \Omega \times x+\rho v\cdot u+\frac{1}{2}\rho ∥v{∥}^{2}. \end{array}$$

The vessel position and velocity and the gravity vector are represented relative to the body frame.

3.5 $$\Gamma =gR^{T}E_{2},\;r=R^{T}q\;\text{and}\;v=R^{T}\dot{q}.$$

With these definitions, the kinetic and potential energies are R-invariant, and there is no explicit dependence on the group action.

Re-introducing integration, the fluid kinetic energy takes the form

$${KE}^{f}=\int_{0}^{L}\int_{0}^{h}\left(\frac{1}{2}\rho ∥u{∥}^{2}+\rho u\cdot \Omega \times x+\rho v\cdot u\right)dy\;dx+\frac{1}{2}\Pi^{f}\Omega \cdot \Omega -m_{f}{\bar{x}}^{f}\cdot \Omega \times v+\frac{1}{2}m_{f}∥v{∥}^{2},$$

where

$$m_{f}=\int_{0}^{L}\int_{0}^{h}\rho \;dy\;dx=\int_{0}^{L}\rho h(x,t)\;dx\;\mathrm{and}\;{\bar{x}}^{f}=\frac{1}{m_{f}}\int_{0}^{L}\int_{0}^{h}\rho x\;dy\;dx,$$

and

$$\Pi^{f}:=\int_{0}^{L}\int_{0}^{h}\rho (1∥x{∥}^{2}-xx^{T})dy\;dx.$$

In the two-dimensional case considered here the moment of inertia term reduces to

3.6 $$\frac{1}{2}\Pi^{f}\Omega \cdot \Omega ={\dot{\theta }}^{2}\int_{0}^{L}\int_{0}^{h}\frac{1}{2}\rho (x^{2}+y^{2})\;dy\;dx=\frac{1}{2}\left[\int_{0}^{L}\left(x^{2}h+\frac{1}{3}h^{3}\right)\;dx\right]{\dot{\theta }}^{2}:=\frac{1}{2}I^{f}{\dot{\theta }}^{2}.$$

The potential energy density for the fluid is

3.7 $$\begin{array}{rl} \hat{{PE}^{f}} & =\rho gE_{2}\cdot (Rx+q)\\ & =\rho gR^{T}E_{2}\cdot (x+R^{T}q)\\ & =\rho \Gamma \cdot (x+r), \end{array}$$

and so, adding in the external pressure field,

$${PE}^{f}=\int_{0}^{L}\int_{0}^{h}\rho \Gamma \cdot (x+r)\;dy\;dx-\int_{0}^{L}\int_{h}^{\infty }p_{a}\;dy\;dx=\Gamma \cdot (m_{f}{\bar{x}}^{f}+m_{f}r)-\int_{0}^{L}\int_{h}^{\infty }p_{a}\;dy\;dx.$$

A similar construction gives the kinetic and potential energies of the vessel

$$\begin{array}{rl} L^{v} & ={KE}^{v}-{PE}^{v}\\ & =\frac{1}{2}\Pi^{v}\Omega \cdot \Omega -m_{v}{\bar{x}}^{v}\cdot \Omega \times v+\frac{1}{2}m_{v}∥v{∥}^{2}-m_{v}\Gamma \cdot ({\bar{x}}^{v}+r), \end{array}$$

where *ρ*_*v*_ is the density of the vessel material,

$$m_{v}=\int_{V}\rho_{v}\;dx\;dy\;\text{and}\;{\bar{x}}^{v}=\frac{1}{m_{v}}\int_{V}\rho_{v}x\;dx\;dy,$$

where $\int_{V}(\cdot )dx\;dy$ is the integral over the vessel volume, and

$$\Pi^{v}\Omega \cdot \Omega :=\left(\int_{V}\rho_{v}(1∥x{∥}^{2}-xx^{T})dx\;dy\;\Omega \right)\cdot \Omega ={\dot{\theta }}^{2}\int_{V}\rho_{v}(x^{2}+y^{2})\;dx\;dy.$$

The full combined Lagrangian, $L=L^{f}+L^{v}$, is

3.8 $$\begin{array}{rl} L & =\int_{0}^{L}\int_{0}^{h}\left(\frac{1}{2}\rho ∥\nabla \psi {∥}^{2}+\rho J^{T}\nabla \psi \cdot \Omega \times x+\rho v\cdot J^{T}\nabla \psi \right)dy\;dx\\ & \;+\frac{1}{2}(\Pi^{f}+\Pi^{v})\Omega \cdot \Omega -(m_{f}{\bar{x}}^{f}+m_{v}{\bar{x}}^{v})\cdot \Omega \times v+\frac{1}{2}(m_{f}+m_{v})∥v{∥}^{2}\\ & \;-\Gamma \cdot (m_{f}{\bar{x}}^{f}+m_{v}{\bar{x}}^{v}+(m_{f}+m_{v})r)+\int_{0}^{L}\int_{h}^{\infty }p_{a}\;dy\;dx, \end{array}$$

where the velocity field has been replaced by its stream function representation

3.9 $$u=J^{T}\nabla \psi \;\mathrm{and}\;J=\left[\begin{array}{cc} 0 & -1\\ 1 & 0 \end{array}\right].$$

In (3.8) *ψ* is any smooth function on D satisfying the boundary conditions

3.10 $$\psi_{y}=0\;\text{at}\;x=0,L,\;\psi_{x}=0\;\text{at}\;y=0\;\text{and}\;\psi_{x}=-h_{t}-\psi_{y}h_{x}\;\text{at}\;y=h.$$

These boundary conditions still leave the freedom to add an arbitrary function of time to *ψ*, and this value is fixed by taking *ψ* = 0 on the rigid boundaries

3.11 ψ(x,0,t)=ψ(0,y,t)=ψ(L,y,t)=0.

When the fluid is neglected and *Γ* is set to zero the Lagrangian density (3.8) reduces to the standard form for a Lagrangian that is left-invariant with respect to the special Euclidean group SE(2), which generates the equations for rigid-body motion undergoing rotation and translation in the plane; see [18], ch. 7 for the theory of SE(*n*)-invariant Lagrangians.

## 4. Variations

In deducing the variational principle in the Eulerian setting, the starting point is the natural variational principle in the LPP setting. In the LPP setting, the principle fluid unknown is the particle position *φ*(**a**, *t*) as defined in (1.1) and its velocity $\dot{\varphi }$ defined in (1.2). In the coupled problem, the orientation R and position **q** are also treated as Lagrangian variables.

The variational principle in the Lagrangian path formulation is therefore

4.1 $$\delta \int_{\tau_{1}}^{\tau_{2}}\hat{L}(\varphi ,\dot{\varphi },R,\dot{R},q,\dot{q})\;d\tau =0,$$

with respect to *free* variations *δφ*, δR, *δ***q**, vanishing at *τ* = *τ*_1_, *τ*_2_, where *τ* = *t* in the LPP setting. The aim is to transform this integrand from Lagrangian variables to Eulerian variables.

The Lagrangian density (3.8) is already a reduced Eulerian form of (4.1) and forms the basis for the variational principle here

$$\delta \int_{t_{1}}^{t_{2}}L(\psi ,h,\Omega ,r,v,\Gamma )\;dt=0,$$

with respect to appropriate *constrained* variations *δψ*, *δh*, *δ****Ω***, *δ***r**, *δ***v**, *δ****Γ***. Taking variations

4.2 $$0=\int_{t_{1}}^{t_{2}}\left[\int_{0}^{L}\int_{0}^{h}\frac{\delta L}{\delta \psi }\delta \psi \;dy\;dx+\int_{0}^{L}\frac{\delta L}{\delta h}\delta h\;dx+\frac{\delta L}{\delta \Omega }\cdot \delta \Omega +\frac{\delta L}{\delta r}\cdot \delta r+\frac{\delta L}{\delta v}\cdot \delta v+\frac{\delta L}{\delta \Gamma }\cdot \delta \Gamma \right]dt.$$

However, setting each of these functional derivatives to zero does not recover the governing equations, since the variations are not free. In this section, the variations needed are recorded and they are justified from the Lagrangian particle path formulation (4.1) in §6. The required fluid variations are

4.3 $$\begin{array}{rl} \delta \psi & =w_{t}+\{w,\psi \}\;(0<y<h,\;0<x<L),\\ \delta \psi & =W_{t}+\psi_{y}{|}^{y=h}\;W_{x}\;(y=h),\\ \delta \psi & =0\;(y=0,\;x=0,L)\\ \;\mathrm{and}\;\delta h & =-W_{x}, \end{array}\}$$

where *w*(*x*, *y*, *t*) is a free variation in the interior with *w*_*y*_ = 0 at *x* = 0, *L*; *w* = 0 at *y* = 0; *w* = 0 at *t* = *t*_1_, *t*_2_, and *W*(*x*, *t*) = *w*(*x*, *h*(*x*, *t*), *t*) is the restriction of *w* to the free surface. As it should, these variations are compatible with the free surface boundary condition (2.12). Indeed, we have

$$\begin{array}{rl} 0 & =\delta (h_{t}+\Psi_{x})={\left(\delta h\right)}_{t}+{\left(\delta \Psi \right)}_{x}\\ & ={\left(\delta h\right)}_{t}+{\left(\delta \psi {|}^{y=h}+\psi_{y}{|}^{y=h}\delta h\right)}_{x}\\ & ={\left(-W_{x}\right)}_{t}+{\left(W_{t}+\psi {|}^{y=h}W_{x}-\psi_{y}{|}^{y=h}W_{x}\right)}_{x}\\ & ={\left(-W_{x}\right)}_{t}+{\left(W_{t}\right)}_{x}=0. \end{array}$$

The vessel variations are

4.4 $$\begin{array}{rl} \delta \Omega & =\Lambda_{t}+\Omega \times \Lambda ,\\ \delta r & =\lambda +r\times \Lambda ,\\ \delta v & =\lambda_{t}+\Omega \times \lambda +v\times \Lambda \\ \;\mathrm{and}\;\delta \Gamma & =\Gamma \times \Lambda , \end{array}\}$$

where ***Λ*** and ***λ*** depend on time only and are free variations vanishing at *t* = *t*_1_, *t*_2_. Substitute these variations into (4.2) noting that ***Ω*** × ***Λ*** = 0 in the two-dimensional case,

$$\begin{array}{rl} 0 & =\int_{t_{1}}^{t_{2}}[\int_{0}^{L}\int_{0}^{h}\frac{\delta L}{\delta \psi }(w_{t}+\{w,\psi \})\;dydx+\int_{0}^{L}\frac{\delta L}{\delta \psi {|}^{y=h}}(W_{t}+uW_{x})\;dx+\int_{0}^{L}\frac{\delta L}{\delta h}(-W_{x})\;dx\\ & \;+\frac{\delta L}{\delta \Omega }\cdot \Lambda_{t}+\frac{\delta L}{\delta r}\cdot (\lambda +r\times \Lambda )+\frac{\delta L}{\delta v}\cdot (\lambda_{t}+\Omega \times \lambda +v\times \Lambda )+\frac{\delta L}{\delta \Gamma }\cdot (\Gamma \times \Lambda )]dt. \end{array}$$

Now include integration over *t*, integrate by parts, and use fixed endpoint conditions on the variations ***Λ*** and ***λ***. The abstract equations emerging are

$$\begin{array}{rl} & \delta w:\frac{D\;}{Dt}\left(\frac{\delta L}{\delta \psi }\right)=0,\\ & \delta W:\frac{\partial \;}{\partial t}\left(\frac{\delta L}{\delta \psi {|}^{y=h}}\right)+\frac{\partial \;}{\partial x}\left(u\frac{\delta L}{\delta \psi {|}^{y=h}}-\frac{\delta L}{\delta h}\right)=0,\;\text{at}\;y=h,\\ & \delta \Lambda :\frac{d}{dt}\left(\frac{\delta L}{\delta \Omega }\right)+r\times \frac{\delta L}{\delta r}+v\times \frac{\delta L}{\delta v}+\Gamma \times \frac{\delta L}{\delta \Gamma }=0\\ \;\mathrm{and}\; & \delta \lambda :\frac{d}{dt}\left(\frac{\delta L}{\delta v}\right)+\Omega \times \frac{\delta L}{\delta v}=\frac{\delta L}{\delta r}. \end{array}$$

The *δw* equation gives the fluid equation in the interior and the *δW* equation generates the GKK conservation law. The *δ****Λ*** and *δ****λ*** equations generate the rigid body motion of the vessel with fluid coupling. The equations are accompanied by the boundary conditions (3.10).

The variational derivatives, obtained by differentiating (3.8), are

4.5 $$\frac{\delta L}{\delta \psi }=-\rho (\Delta \psi -2\dot{\theta }),\;0<y<h,$$

in the interior, and at the free surface

4.6 $$\frac{\delta L}{\delta \psi }=K\;\text{and}\;\frac{\delta L}{\delta h}=\Xi \;\text{at }y=h.$$

A derivation of these variations starting with (3.8) is given in appendix A.

The variational derivatives associated with the rigid body motion are

4.7 $$\begin{array}{rl} \frac{\delta L}{\delta \Omega } & =(\Pi^{f}+\Pi^{v})\Omega +(m_{f}{\bar{x}}^{f}+m_{v}{\bar{x}}^{v})\times v+\int_{0}^{L}\int_{0}^{h}(x\times \rho u)\;dy\;dx,\\ \frac{\delta L}{\delta r} & =-(m_{f}+m_{v})\Gamma ,\\ \frac{\delta L}{\delta v} & =(m_{f}+m_{v})v+\Omega \times (m_{f}{\bar{x}}^{f}+m_{v}{\bar{x}}^{v})+\int_{0}^{L}\int_{0}^{h}\rho u\;dy\;dx\\ \;\mathrm{and}\;\frac{\delta L}{\delta \Gamma } & =-g(m_{f}{\bar{x}}^{f}+m_{v}{\bar{x}}^{v}+(m_{f}+m_{v})r). \end{array}\}$$

Substituting these expressions into the *δ****Λ*** and *δ****λ*** equations gives the equations for the vessel coupled to the fluid motion, and they recover exactly the conservation of total linear and angular momentum in §2c. Here the governing equation for **v** is expanded

4.8 $$\begin{array}{rl} & \frac{d}{dt}\left((m_{f}+m_{v})v+\Omega \times (m_{f}{\bar{x}}^{f}+m_{v}{\bar{x}}^{v})+\int_{0}^{L}\int_{0}^{h}\rho u\;dy\;dx\right)\\ & \;+\Omega \times \left((m_{f}+m_{v})v+\Omega \times (m_{f}{\bar{x}}^{f}+m_{v}{\bar{x}}^{v})+\int_{0}^{L}\int_{0}^{h}\rho u\;dy\;dx\right)\\ & \;=-(m_{f}+m_{v})\Gamma , \end{array}$$

where **u** = (*ψ*_*y*_, − *ψ*_*x*_). This expression generates only two component equations as the third component is identically zero. A similar expanded formula can be developed for the ***Ω*** equation, with the ***Ω*** equation having only one non-zero component. When the velocity field **u** is restricted to be irrotational, the vessel equations agree with those in [9,10].

## 5. Special cases of governing equations

When the vessel motion vanishes and *p*_*a*_ = 0, the Lagrangian (3.8) reduces to

$$L=\int_{0}^{L}\int_{0}^{h}\left(\frac{1}{2}\rho ∥\nabla \psi {∥}^{2}-\rho gy\right)\;dy\;dx,$$

with

$$\delta L=\int_{0}^{L}\int_{0}^{h}\rho \nabla \psi \cdot \nabla \delta \psi \;dy\;dx+\int_{0}^{L}{\left[\frac{1}{2}\rho ∥\nabla \psi {∥}^{2}-\rho gy]\right|}^{y=h}\delta h\;dx.$$

Substituting for *δψ* and *δh* from (4.3), and adding in the boundary conditions (3.10), recovers the governing equations for the fluid in §2b with the GKK conservation law in the form (2.18).

When the fluid motion vanishes, the vessel motion equations reduce to

5.1 $$\begin{array}{rl} & \Pi^{v}\Omega_{t}+m_{v}{\bar{x}}^{v}\times v_{t}+m_{v}(v\cdot {\bar{x}}^{v})\Omega -m_{v}\Gamma \times {\bar{x}}^{v}=0,\\ & m_{v}v_{t}+m_{v}(\Omega \times v)+m_{v}(\Omega_{t}\times {\bar{x}}^{v})+m_{v}\Gamma -m_{v}\Omega \cdot \Omega {\bar{x}}^{v}=0\\ \;\mathrm{and}\; & \Gamma_{t}+\Omega \times \Gamma =0. \end{array}\}$$

**Figure 2.**
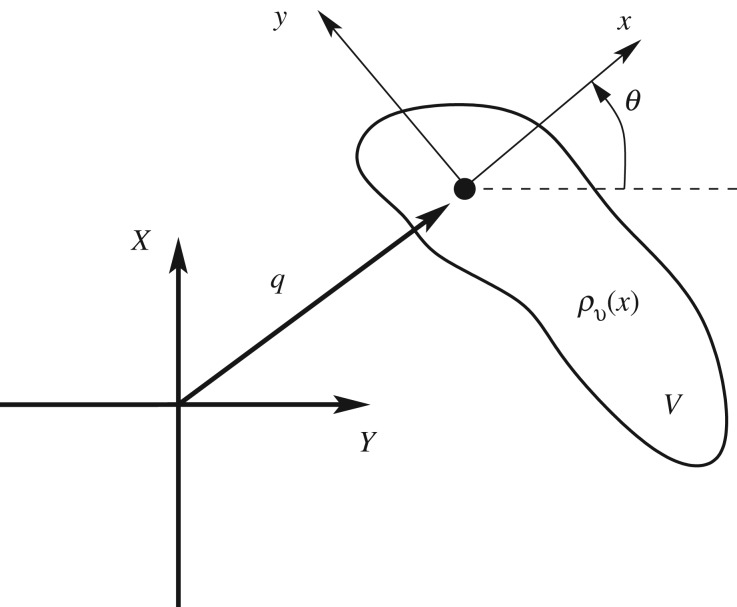
Schematic of rigid body motion in a vertical plane.

These equations are similar to Kirchoff's equations for a rigid body in moving in 3D (see Lamb [19, ch. 6] and Holm [18, §7.2]). The latter view of the equations (5.1) is that they are the Euler–Poincaré equations for a Lagrangian that is left-invariant with respect to the group SE(2), the special Euclidean group in the plane, although here there is the additional term due to gravity and represented by the vector ***Γ***. In two dimensions, these equations are just the governing equations of a compound pendulum in the plane with translating pivot point (figure 2), or more generally a rigid body moving in the plane subject to a gravitational field. In two dimensions, the first equation of (5.1) has only one non-zero component and the second has only two non-zero components.

## 6. Justifying constrained variations via reduction

The natural variational principle in fluid mechanics is Hamilton's principle in the LPP setting. Natural because the density is the kinetic minus potential energy and the variations are free. In the LPP setting, the position of particles is determined by a mapping φ:B→D where *B* is a reference space

$$B=\{a=(a_{1},a_{2})\in R^{2}:0\leq a_{1}\leq 1,\;0\leq a_{2}\leq 1\}$$

and D is the current fluid configuration (2.1). The position and velocity of a fluid particle at any time *τ* in the LPP setting from (1.1) and (1.3) are recorded here,

6.1 $$x=\varphi (a,\tau )\;\text{and}\;u^{Lag}(a,\tau )=\dot{\varphi }(a,\tau ).$$

*τ* = *t* but the distinction is maintained as ∂_*τ*_ is taken with **a** fixed and ∂_*t*_ is taken with **x** fixed.

The LPP Lagrangian variational principle for the fluid only is then

6.2 $$\delta \int_{\tau_{1}}^{\tau_{2}}L(\varphi ,\dot{\varphi })\;d\tau =0,$$

with respect to arbitrary variations *δφ* vanishing at *τ* = *τ*_1_, *τ*_2_. To be precise the configuration manifold is an appropriate subset of the manifold of volume-preserving embeddings (e.g. [15,20,21] and references therein) but this level of detail will not be needed here.

The free surface is the image of the upper edge of the reference space

6.3 $$\Sigma^{s}=\{(x,y)\in R^{2}:x=\varphi_{1}(a_{1},1,\tau )\;\text{and}\;y=\varphi_{2}(a_{1},1,\tau )\}\subset \partial D.$$

Variation of L in (3.8) will require variations in the Eulerian setting. The transformation from the LPP description to the Eulerian description is a form of reduction, obtained by factoring out the particle relabelling group (cf. §11.1 of [13,15] when a free boundary is included). This reduction converts free variations in the LPP setting to constrained variations in the Eulerian setting.

The Lagrangian and Eulerian velocity fields are related by

6.4 $$u(x,t)=\dot{\varphi }(\varphi^{-1}(x,t),t)\Rightarrow \dot{\varphi }(a,\tau )=u(\varphi (a,\tau ),\tau ),$$

and **u** is a divergence-free vector field on D, parallel to rigid boundaries but not parallel to *Σ*^*s*^. Let *δφ*(**a**, *τ*) be a free variation of the fluid displacement in the LPP setting and let **z**(**x**, *t*) be its representation in the Eulerian setting,

6.5 $$z(x,t)=\delta \varphi (\varphi^{-1}(x,t),t)\Rightarrow \delta \varphi (a,\tau )=z(\varphi (a,\tau ),\tau ).$$

The field **z** is an arbitrary divergence-free vector field on D, parallel to rigid boundaries but not parallel to *Σ*^*s*^.

To derive (1.5) vary (6.4),

6.6 $$\delta \dot{\varphi }(a,\tau )=\delta u(\varphi (a,\tau ),\tau )+\delta \varphi (a,\tau )\cdot \nabla u(\varphi (a,\tau ),\tau ).$$

Now differentiating (6.5) with respect to *t* gives

6.7 $$\delta \dot{\varphi }(a,\tau )=z_{t}(\varphi (a,\tau ),\tau )+\dot{\varphi }(a,\tau )\cdot \nabla z(\varphi (a,\tau ),\tau ).$$

Combining these two expressions then gives

6.8 $$\delta u=z_{t}+[u,z]\;\text{with}\;[u,z]:=u\cdot \nabla z-z\cdot \nabla u.$$

In forming this equation, **a** is replaced by **a** = *φ*^−1^(**x**, *t*) rendering it a purely Eulerian expression. The identity (6.8) is one of the most important, but unheralded, identities in fluid mechanics as it relates small changes in the Eulerian velocity field to small changes in the Lagrangian velocity field, all viewed from the Eulerian perspective. Further detail on this identity, its history, abstraction and generalization can be found in [13,14] and references therein.

The two new results on Eulerian variations needed in this paper are the implications for (6.8) when the velocity field is represented by a stream function, and the induced free surface variation when the surface *Σ*^*s*^ is represented by a graph.

### (a) Stream function variations

The aim is to reduce

6.9 $$\delta u=z_{t}+[u,z]\;\text{to}\;\delta \psi =w_{t}+\{w,z\}.$$

Define *w*(*x*, *y*, *t*) by expressing the divergence-free vector field **z** in terms of a *w* stream function

6.10 $$z:=J^{T}\nabla w.$$

The properties of **z** then give that *w* is an arbitrary scalar-valued function on D satisfying *w*_*y*_ = 0 at *x* = 0, *L*; *w*_*x*_ = 0 at *y* = 0 and *w* = 0 at *t* = *t*_1_, *t*_2_.

The *w* stream function is obtained from a given **z** by integrating

$$dw=-z_{2}\;dx+z_{1}\;dy,$$

along a curve in D. By choosing a reference value of *w*, e.g. fixing *w* on rigid boundaries

6.11 w(x,0,t)=w(0,y,t)=w(L,y,t)=0,

the *w* stream function is unique. This condition mirrors the condition on *ψ* in (3.11).

Inserting the velocity representation **u** = **J**^T^∇*ψ* and (6.10) into the first expression in (6.9) gives

6.12 $$J^{T}\nabla \delta \psi =J^{T}\nabla w_{t}+[J^{T}\nabla \psi ,J^{T}\nabla w].$$

A direct computation then shows that [**J**^T^∇*ψ*, **J**^T^∇*w*] = **J**^T^∇{*w*, *ψ*}, and so (6.12) yields

$$J^{T}\nabla (\delta \psi -w_{t}-\{w,\psi \})=0$$

or

6.13 $$\delta \psi =w_{t}+\{w,\psi \}+f(t),$$

where *f*(*t*) is in general arbitrary. However, evaluating (6.13) at *y* = 0 and using the normalization (6.11) gives *f*(*t*)=0. This confirms the form for *δψ* in the second expression in (6.9).

### (b) Free surface variations

With the free surface represented by a graph, *y* = *h*(*x*, *t*), the mapping from LPP to Eulerian variables (6.3) on *Σ*^*s*^ becomes

6.14 $$h(\varphi_{1}(a_{1},1,\tau ),\tau )=\varphi_{2}(a_{1},1,\tau ).$$

The time derivative of this equality gives the free surface condition (2.5). Taking variations of (6.14), we get

6.15 $$\delta h(\varphi_{1}(a_{1},1,\tau ),\tau )+h_{x}\delta \varphi_{1}(a_{1},1,\tau )=\delta \varphi_{2}(a_{1},1,\tau ).$$

But using the stream function representation for **z**, we get

$$\delta \varphi (a,\tau )=z(\varphi (a,\tau ),\tau )=(w_{y}(\varphi (a_{1},a_{2},\tau ),\tau ),-w_{x}(\varphi (a_{1},a_{2},\tau ),\tau ))$$

and hence

$$\delta \varphi_{1}(a_{1},1,\tau )=w_{y}(\varphi (a_{1},1,\tau ),\tau )\;\text{and}\;\delta \varphi_{2}(a_{1},1,\tau )=-w_{x}(\varphi (a_{1},1,\tau ),\tau ),$$

so the second and third terms in (6.15) combine into

$$h_{x}\delta \varphi_{1}(a_{1},1,\tau )-\delta \varphi_{2}(a_{2},1,\tau )=h_{x}w_{y}(\varphi_{1}(a_{1},1,\tau ),1,\tau )+w_{x}(\varphi_{1}(a_{1},1,\tau ),1,\tau ).$$

In Eulerian variables, the right-hand side is

$$[h_{x}w_{y}(\varphi_{1},1,\tau )+w_{x}(\varphi_{1},1,\tau )]{|}^{a_{2}=1}\overset{\varphi^{-1}}{\to }h_{x}w_{y}(x,h(x,t),t)+w_{x}(x,h(x,t),t)=W_{x}.$$

Substitution into (6.15) and mapping *δh* to Eulerian variables then gives

$$\delta h=-W_{x},$$

confirming the expression in (4.3).

### (c) Variation of vessel parameters

The variations of ***Ω***, **r**, **v** and ***Γ*** in (4.4) arise in rigid body motion and the details can be found in [13]. Here, just a sketch of the basic idea is given. A detailed derivation of *δ****Ω*** is given on pages 249–250 of [13] with $\hat{\Lambda }:=R^{T}\delta R$, where $\hat{\Lambda }$ is the representation of the vector ***Λ*** in terms of a 3 × 3 skew-symmetric matrix. Let $\lambda =R^{T}\delta q$ then

$$\delta r=\delta (R^{T}q)=-R^{T}\delta RR^{T}q+R^{T}\delta q=\lambda -\hat{\Lambda }r=\lambda +r\times \Lambda .$$

A similar argument gives the expression for *δ***v** in (4.4). For the gravity vector

$$\delta \Gamma =\delta (R^{T}e_{2})=-R^{T}\delta RR^{T}e_{2}=-R^{T}\delta R\Gamma =-\Lambda \times \Gamma ,$$

confirming the fourth equation in (4.4).

## 7. Implications and applications

The existence of a variational principle for sloshing with vorticity gives added value to the analysis of the governing equations in various directions. For example, the importance of variational principles in the design of robust numerical schemes is now well established (e.g. [22,23]). In fluid dynamics, the development of numerical methods from variational principles with constrained variations, as in the Euler–Poincaré framework, is a burgeoning research area (cf. Pavlov *et al.* [24] and its citation trail). For this development, the understanding of the Eulerian variational principle as a consequence of the LPP is of fundamental importance. The variational framework in the present paper is a candidate for adapting these variational numerical methods to the case of free surface flows.

**Figure 3.**
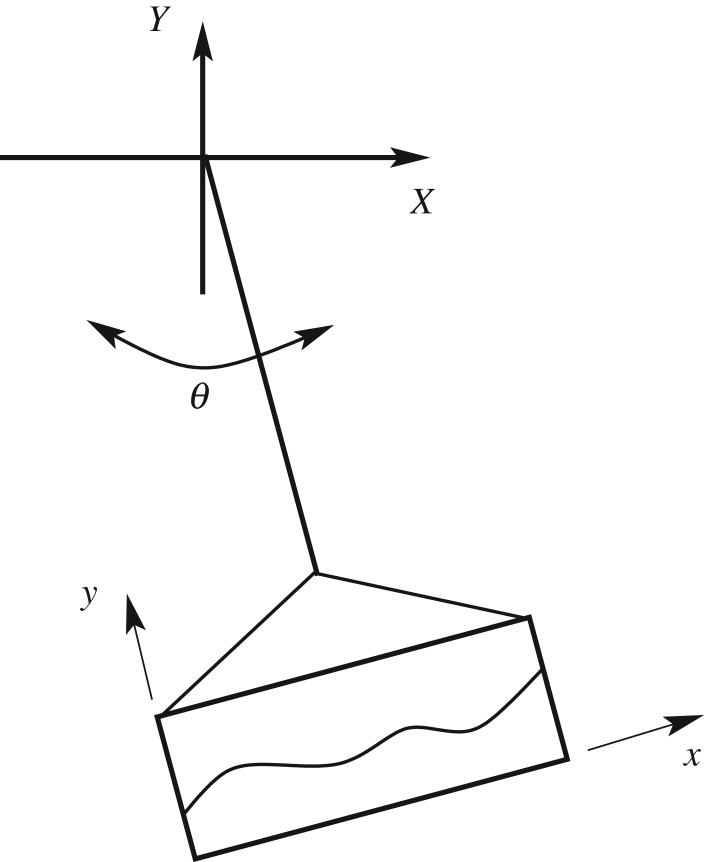
The pendulum slosh problem with vorticity.

A canonical problem in the study of dynamic coupling between sloshing and vessel motion is the ‘pendulum-slosh’ problem, illustrated in figure 3, where **q** = 0 and the only vessel degree of freedom is rotation. The irrotational case has been studied in Turner *et al*. [25]. Vorticity will change the resonance structure, affect the energy budget and interact with the vorticity induced by vessel rotation. An analysis of this problem is outside the scope of this paper, but some indication of the implication of vorticity on sloshing can be given using an exact solution.

There is an exact solution of the sloshing problem with vorticity and a stationary vessel. Suppose the external pressure field evaluated on the free surface has the form

$$p_{a}{|}^{y=h}=\frac{1}{4}\rho \beta^{2}A\cos(2\alpha x),$$

where *α*, *β*, *A* are arbitrary positive parameters, then the exact steady solution, with a non-trivial vorticity field, is

7.1 $$\begin{array}{rl} u(x,y) & =\beta_{m}A\sin(\alpha_{n}x)\cos(\beta_{m}y),\\ v(x,y) & =-\alpha_{n}A\cos(\alpha_{n}x)\sin(\beta_{m}y),\\ p(x,y) & =\frac{1}{4}\rho \beta_{m}^{2}A\cos(2\alpha_{n}x)-\rho gy+\frac{1}{4}\rho \alpha_{n}^{2}A\cos(2\beta_{m}y)+Const.\\ \;\mathrm{and}\;V(x,y) & =(\alpha_{n}^{2}+\beta_{m}^{2})A\sin(\alpha_{n}x)\sin(\beta_{m}y), \end{array}\}$$

with *h* = *h*_0_ with *h*_0_ a constant, and

$$\alpha_{n}=\frac{n\pi }{L}\;\text{and}\;\beta_{m}=\frac{m\pi }{h_{0}},$$

where *m*, *n* are natural numbers. The constant in the pressure is chosen to satisfy the free surface boundary condition giving

$$Const.=\rho gh_{0}-\frac{1}{4}\rho \alpha_{n}^{2}A\cos(2\beta_{m}h_{0}).$$

This exact solution has not been noticed before as it requires a non-zero external pressure field. It is useful as a model problem for introducing vorticity. It also demonstrates the importance of non-zero kinetic energy in the initial data when vorticity is present. The kinetic energy of the fluid in this case is

7.2 $${KE}^{f}=\int_{0}^{L}\int_{0}^{h_{0}}\frac{1}{2}(u^{2}+v^{2})\;dy\;dx=\frac{1}{8}\pi^{2}A^{2}\left(\frac{n^{2}}{L^{2}}+\frac{m^{2}}{h_{0}^{2}}\right).$$

**Figure 4.**
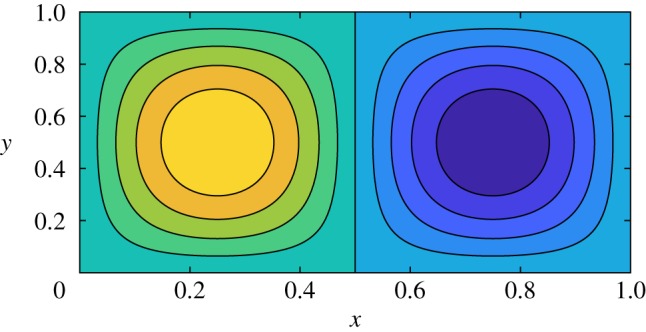
Stream function contours for the solution (7.1) in the case *n* = 2 and *m* = 1. (Online version in colour.)

The flowfield has a cellular structure with *n* cells in the *x*-direction and *m* cells in the *y*-direction. An example of the stream function contours in the case *n* = 2, *m* = 1 is shown in figure 4. Although the flow is stationary, the formula (7.2) shows that the kinetic energy increases with granularity of the vorticity. This cellular structure is a model for the vorticity generated when baffles are introduced. Adding a perturbation to this exact solution, and using it as initial data, is a mechanism for generating a non-trivial sloshing flow field with time-dependent vorticity.

Some experiments show evidence of vorticity. In a viscous fluid, vorticity can be generated by boundary layers and vortex shedding. For short times, it is the vorticity in initial data that will dominate. Many sloshing experiments start with a quiescent fluid (e.g. [26]), generating an irrotational flow until viscosity influences the vorticity field. However, there are some interesting experiments of Herczyńiski & Weidman [27], which introduce non-zero kinetic energy into the initial data. In [27], they were interested in the induced motion of the vessel due to the fluid. The fluid was set in motion and transients eliminated before recording, thereby ensuring non-trivial kinetic energy in the initial data. Vorticity was not measured and the experiments were compared with irrotational theory, but the experiment shows that it is possible to generate initial conditions with vorticity in a sloshing experiment. Another approach to generating strong vorticity in the initial data is to insert a baffle to generate vortex shedding before starting the experimental record.

## 8. Concluding remarks

In this paper, a new variational principle has been introduced for two-dimensional inviscid incompressible fluid flow in a moving vessel with coupling to the vessel motion. It is a starting point for new applications and new directions (three-dimensionality, other geometries, multi-valued free surface and effects such as surface tension).

The representation of the surface as a graph, *y* = *h*(*x*, *t*) simplified the analysis, but the variational principle can be generalized to the case where the surface is represented parametrically, *x* = *X*(*s*, *t*) and *y* = *Y* (*s*, *t*), for 0 < *s* < *L*, thereby allowing for overhanging and a multi-valued free surface. Coupling with elastic vessel motion or elastic pendulum rod is also a potential generalization. Sloshing in microgravity can be modelled by introducing surface tension which fits neatly into the variational framework since surface tension is intrinsically linked to the variational derivative of the free surface mean curvature.

The Lagrangian variational principle here opens the door to generating a Hamiltonian formulation with a Lie–Poisson structure, extending the theory in Lewis *et al.* [28] (for incompressible free surface flows) and Mazer & Ratiu [29] (for compressible free surface flows) to the dynamically coupled problem.

In the formulation presented here, there were hints about the structure of the problem in 3D. However there are still technical difficulties in extending the fluid part to 3D. Firstly, a parametrization of divergence-free vector fields is required. The preferred option is a vector stream function, but the pressure boundary condition then presents challenges. The tangential derivative of pressure has two components and the extension of the GKK theory to 3D does not result in a conservation law (see [16] for the GKK theory in 3D without rotation). On the other hand, the extension of the vessel motion to 3D is straightforward.

Three-dimensional cylindrical geometry has many applications. Timokha [5] discusses vorticity in sloshing, and comments on the ‘glass-wine’ paradox, whereby a steady-state swirl motion in a vessel generates a vortex by conversion of the wave angular momentum to the vortex angular momentum. Modelling propellant sloshing in rockets also requires cylindrical geometry in 3D. Another interesting application of cylindrical geometry is the ignoble problem of ‘walking with coffee’ (cf. Mayer & Krechetnikov [30]). In [30], a simple mechanical model is developed for the interaction between sloshing coffee and the cup's dynamics and they comment on the possibility of designing a flexible container to suppress the sloshing. In addition to flexibility, it may be that swirl or vorticity or the variational structure also play a role.

## Data accessibility

This article has no additional data.

## Appendix A. Details of the δL calculations

First look at the variations associated with the stream function with the other variables fixed. The stream function terms in L are

$$L^{\psi }=\int_{0}^{L}\int_{0}^{h}\left(\frac{1}{2}\rho (\psi_{x}^{2}+\psi_{y}^{2})-\rho \dot{\theta }(x\psi_{x}+y\psi_{y})+\rho (v_{1}\psi_{y}-v_{2}\psi_{x})\right)dy\;dx.$$

Vary $L^{\psi }$ with respect to *ψ* keeping all other variables fixed, integrate by parts and use *δψ* = 0 on rigid boundaries from (3.10), then

$$\begin{array}{rl} \delta L^{\psi } & =-\int_{0}^{L}\int_{0}^{h}\rho (\Delta \psi -2\dot{\theta })\delta \psi \;dy\;dx+\int_{0}^{L}\rho (\psi_{y}-\dot{\theta }h+v_{1})\delta \psi {|}^{y=h}\;dx\\ & \;+\int_{0}^{L}\rho h_{x}(-\psi_{x}+\dot{\theta }x+v_{2})\delta \psi {|}^{y=h}\;dx\\ & =-\int_{0}^{L}\int_{0}^{h}\rho (\Delta \psi -2\dot{\theta })\delta \psi \;dy\;dx+\int_{0}^{L}K\;\delta \psi {|}^{y=h}\;dx. \end{array}$$

This confirms the variational derivatives (4.5) and the first of (4.6).

Now keep all other variations fixed, and vary with respect to *h*,

$$\begin{array}{rl} \delta L & =\int_{0}^{L}[\left(\frac{1}{2}\rho ∥u{∥}^{2}+\rho u\cdot \Omega \times x+\rho v\cdot u\right)\delta h+\frac{1}{2}\rho (x^{2}+h^{2})\Omega \cdot \Omega \delta h\\ & \;-\rho (x{|}^{y=h})\cdot \Omega \times v\delta h+\frac{1}{2}\rho \delta h∥v{∥}^{2}\delta h-\rho \Gamma \cdot (x{|}^{y=h}+r)\delta h-p_{a}{|}^{y=h}\delta h\;]dx, \end{array}$$

using

$$\delta m_{f}=\int_{0}^{L}\rho \delta h\;dx,\;\delta (m_{f}{\bar{x}}^{f})=\int_{0}^{L}\rho x{|}^{y=h}\delta h\;dx\;\text{and}\;\delta \int_{0}^{h}\frac{1}{2}\rho (x^{2}+y^{2})\;dy=\frac{1}{2}\rho (x^{2}+h^{2})\delta h.$$

Rearranging and substituting the above expressions

A 1 $$\frac{\delta L}{\delta h}=\frac{1}{2}\rho {\left(u-\dot{\theta }h+v_{1}\right)}^{2}+\frac{1}{2}\rho {\left(v+\dot{\theta }x+v_{2}\right)}^{2}-\rho (\Gamma_{1}(x+r_{1})+\Gamma_{2}(h+r_{2}))-p_{a}{|}^{y=h},$$

which confirms the second of (4.6) using (2.17).

For the variations associated with vessel motion, keep *ψ* and *h* fixed and vary with respect to ***Ω***, **r**, **v** and ***Γ***,

$$\begin{array}{rl} \delta L & =\int_{0}^{L}\int_{0}^{h}(\rho u\cdot \delta \Omega \times x+\rho \;\delta v\cdot u)dy\;dx+(\Pi^{f}+\Pi^{v})\Omega \cdot \delta \Omega \\ & \;-(m_{f}{\bar{x}}^{f}+m_{v}{\bar{x}}^{v})\cdot (\delta \Omega \times v+\Omega \times \delta v)+(m_{f}+m_{v})v\cdot \delta v\\ & \;-(m_{f}+m_{v})\Gamma \cdot \delta r-\delta \Gamma \cdot (m_{f}{\bar{x}}^{f}+m_{v}{\bar{x}}^{v}+(m_{f}+m_{v})r). \end{array}$$

Rearrange and group terms

$$\begin{array}{rl} \delta L & =\delta \Omega \cdot \int_{0}^{L}\int_{0}^{h}(\rho x\times u)\;dy\;dx+\delta \Omega \cdot (m_{f}{\bar{x}}^{f}+m_{v}{\bar{x}}^{v})\times v+(\Pi^{f}+\Pi^{v})\Omega \cdot \delta \Omega \\ & \;+\delta v\cdot \int_{0}^{L}\int_{0}^{h}\rho u\;dy\;dx+\Omega \times (m_{f}{\bar{x}}^{f}+m_{v}{\bar{x}}^{v})\cdot \delta v+(m_{f}+m_{v})v\cdot \delta v\\ & \;-(m_{f}+m_{v})\Gamma \cdot \delta r-\delta \Gamma \cdot (m_{f}{\bar{x}}^{f}+m_{v}{\bar{x}}^{v}+(m_{f}+m_{v})r). \end{array}$$

Extracting the variational derivatives,

$$\delta L=\frac{\delta L}{\delta \Omega }\cdot \delta \Omega +\frac{\delta L}{\delta v}\cdot \delta v+\frac{\delta L}{\delta r}\cdot \delta r+\frac{\delta L}{\delta \Gamma }\cdot \delta \Gamma ,$$

confirms the formulae in (4.7).
